# Developmentally Regulated Modulation of Lumbar Motoneurons by Metabotropic Glutamate Receptors: A Cellular and Behavioral Analysis in Newborn Mice

**DOI:** 10.3389/fncel.2021.770250

**Published:** 2021-12-09

**Authors:** Camille Quilgars, Jean-René Cazalets, Sandrine S. Bertrand

**Affiliations:** ^1^Institut de Neurosciences Cognitives et Intégratives d’Aquitaine (INCIA), CNRS UMR 5287, Université de Bordeaux, Bordeaux, France; ^2^Ecole Pratique des Hautes Etudes (EPHE), Paris Sciences et Lettres University, Paris, France

**Keywords:** spinal cord, motoneuron (MN), mGlu receptors, synaptic plasticity, development

## Abstract

The present study explores the impact of metabotropic glutamate receptor (mGluR) activation on activity-dependent synaptic plasticity (ADSP) and the intrinsic membrane properties of lumbar motoneurons (MNs) using a combination of biochemical, pharmacological, electrophysiological and behavioral techniques. Using spinal cord slices from C57BL/6JRJ mice at two developmental stages, 1-3 and 8-12 postnatal days (P1-P3; P8-P12, respectively), we found that ADSP expressed at glutamatergic synapses between axons conveyed in the ventrolateral funiculus (VLF) and MNs, involved mGluR activation. Using specific agonists of the three groups of mGluRs, we observed that mGluR stimulation causes subtype-specific and developmentally regulated modulation of the ADSP and synaptic transmission at VLF-MN synapses as well as the intrinsic membrane properties of MNs. RT-qPCR analysis revealed a downregulation of mGluR gene expression with age in the ventral part of the lumbar spinal cord. Interestingly, the selective harvest by laser microdissection of MNs innervating the *Gastrocnemius* and *Tibialis anterior* muscles unraveled that the level of Grm2 expression is higher in *Tibialis* MNs compared to *Gastrocnemius* MNs suggesting a specific mGluR gene expression profile in these two MN pools. Finally, we assessed the functional impact of mGluR modulation on electrically induced bouts of fictive locomotion in the isolated spinal cord preparation of P1-P3 mice, and *in vivo* during spontaneous episodes of swimming activity in both P1-P3 and P8-P12 mouse pups. We observed that the mGluR agonists induced distinct and specific effects on the motor burst amplitudes and period of the locomotor rhythms tested and that their actions are function of the developmental stage of the animals. Altogether our data show that the metabotropic glutamatergic system exerts a complex neuromodulation in the developing spinal lumbar motor networks and provide new insights into the expression and modulation of ADSP in MNs.

## Introduction

Glutamate, the main excitatory neurotransmitter of the central nervous system (CNS) acts via four types of ionotropic receptors (N-methyl-D-aspartate (NMDA), α-amino-3-hydroxy-5-methyl-4-isoxazolepropionic acid (AMPA), Kainate and Delta receptors) (Madden, 2002; Traynelis et al., 2010) and eight metabotropic glutamate receptors (mGluRs) (Niswender and Conn, 2010; Reiner and Levitz, 2018). mGluRs can be subdivided into three different families based on sequence homology, G-protein coupling and pharmacological profile: the group I mGluR (mGluR1 and mGluR5) coupled to Gq/11 protein and the mGluR II (mGluR2 and 3) and mGluR III (mGluR4, 7 and 8) associated to Gi/o protein. With the exception of mGluR6, whose localization is restricted to the retina, all mGluRs are ubiquitously expressed throughout the CNS with some subtype specificity in different anatomical regions. mGluRs are present at both post- and presynaptic levels in neurons as well as in astrocytes, and play key roles in the control of neuronal excitability, synaptic release and activity-dependent synaptic plasticity (ADSP) (Niswender and Conn, 2010; Atwood et al., 2014; Reiner and Levitz, 2018; Crupi et al., 2019). mGluR involvement in ADSP has been extensively studied in brain structures (Gubellini et al., 2004; Gladding et al., 2009; Collingridge et al., 2010; Lüscher and Huber, 2010; Atwood et al., 2014; Crupi et al., 2019), where long term depression (LTD) linked to the different mGluR subtypes has been described (Gladding et al., 2009; Atwood et al., 2014) as well as the mandatory role of glutamate spillover and subsequent activation of perisynaptic mGluRs for the induction of long-term potentiation (LTP) (Gonçalves-Ribeiro et al., 2019; Valtcheva and Venance, 2019). In the spinal cord, mGluRs are densely expressed in the dorsal horn where they actively participate in the integration of noxious and non-noxious inputs in sensory networks (Goudet, 2018). In the ventral spinal cord, numerous studies have provided detailed information on mGluR-mediated effects, especially mGluR I, on the different cellular components of the motor spinal circuits as well as on the motor activities they generate, using *in vitro* spinal cord preparations from several species such as lampreys, Xenopus and newborn rodents (Krieger et al., 1996, 1998, 2000; Kettunen et al., 2002, 2003; Krieger and Manira, 2002; Marchetti et al., 2003, 2005; Taccola et al., 2003, 2004b; Nistri, 2006; Chapman and Sillar, 2007; Kyriakatos and Manira, 2007; Iwagaki and Miles, 2011).

Linked to intracellular calcium signaling, mGluRs play important roles in many aspects of CNS maturation and are known to display developmentally regulated expression patterns with highest global levels reported in the neonate and juvenile brain and spinal cord (Catania et al., 1994; Berthele et al., 1999; Hubert and Smith, 2004). Moreover, developmental alteration in ADSP in supraspinal structures have been correlated with changes in mGluR subtype expression (Grassi et al., 2002; Puyal, 2003; Falcón-Moya et al., 2020). We have previously shown that ADSP can be expressed at synapses between axons conveyed in the ventrolateral funiculus (VLF) of the spinal cord and lumbar motoneurons (MNs) after high frequency stimulation of the VLF and that this ADSP varies according to both the developmental stage and flexor or extensor function of MNs (Lenschow et al., 2016). Therefore, we asked whether mGluRs participate in ADSP in developing MNs, and more globally, whether mGluR-mediated modulation is developmentally regulated in lumbar MNs. Here, using newborn mice and specific agonists of the different mGluR groups, we demonstrate that (1) mGluRs are involved in ADSP expression at VLF-MN synapses, (2) both ADSP and MN intrinsic membrane properties are differentially and developmentally modulated by the different mGluR subtypes, and (3) MNs exhibit a particular mGluR expression pattern depending on whether they are flexor or extensor related neurons. Finally, we assessed the functional impact of mGluR modulation both *in vitro* in the isolated spinal cord preparation and *in vivo* in behaving mouse pups during swimming.

## Materials and Methods

### Animals and Ethics

Experiments were performed on 326 new-born C57BL/6JRJ mice, aged postnatal day 1 (P1) to P3 and P8 to P12, without consideration of sex. All procedures were carried out in strict accordance with the guidelines of the European Committee Council Directive and approved by the local ethic committee of the University of Bordeaux and the French Agriculture and Forestry Ministry for handling animals (approval number 2016012716035720). All efforts were made to minimize animal suffering and reduce the number of animals used.

### *In vitro* Spinal Cord Preparation

1-3 postnatal days mouse pups were anesthetized with 4% isoflurane until reflexes were lost and eviscerated after decapitation. Spinal cords were isolated via laminectomy, sectioned at the thoracic level 1 (T1) and placed ventral side up in the recording chamber. Dissection and recording procedures were performed under continuous superfusion with artificial cerebrospinal fluid (aCSF) containing the following (in mM): NaCl 130; KCl 3; CaCl_2_ 2.50; MgSO_4_ 1.3; NaH_2_PO_4_ 0.58; NaHCO_3_ 25; glucose 10; equilibrated with 95% O_2_, 5% CO_2_ adjusted to pH 7.4 at room temperature (23°C). Recordings were performed after a recovery period of at least one hour.

### Spinal Cord Slices

Lumbar spinal cords from P1-P3 and P8-P12 mice were dissected free in an ice-cold sucrose-based saline solution containing the following (in mM): KCl 2, CaCl_2_ 0,5, MgCl_2_ 7, NaH_2_PO_4_ 1,15, NaHCO_3_ 26, glucose 11 and sucrose 205, bubbled with 95% O_2_, 5% CO_2_ and sectioned into transverse slices (350 μm) with a vibroslicer (Leica, VT1000S). Slices were allowed to recover in oxygenated aCSF (see above) for at least 1 h at 30°C before being transferred to the recording chamber.

### Extracellular Recordings

In the *in vitro* spinal cord preparation, motor output was recorded extracellularly using a custom-made differential AC amplifier from the right and left lumbar 2 (rL2, lL2, respectively), rL5 and lL5 ventral roots using glass suction electrodes filled with aCSF. Bouts of fictive locomotion were evoked by electrical stimulation (50 Hz, 2 s) of the VLF using a concentric bipolar electrode (Phymep) positioned between the T9 and T11 levels (Magnuson and Trinder, 1997). Neurograms were amplified (x 5000) using high impedance AC amplifiers (200 – 3000 Hz) built at the laboratory, digitized, acquired at 2 kHz and integrated using Axograph software (Axograph, Australia), for future analysis. Ten VLF stimulations were applied with an interval of 2 min. The stimulation series was started at the beginning of the perfusion of the mGluR agonists. In our experimental conditions, with a flow rate of 7 ml/min, the solution in the bath was completely replaced around 10 min after the beginning of the perfusion. To reduce the number of animals used, after a first series of VLF stimulations performed in aCSF, the three agonists were successively applied always in the same order (DHPG, LY354740 and then L-AP4) on each spinal cord preparation tested, with a wash-out period of at least one hour with aCSF between each drug perfusion. To allow comparison between experiments and drug applications, values of motor burst amplitudes and rhythm periods were normalized to the mean amplitude and period, respectively, measured during the first two responses obtained in a given series.

### Intracellular Recordings

Whole-cell patch clamp recordings from putative MNs located throughout the lumbar spinal cord enlargement, identified by their relatively large soma (superior to 20 μm) and location in the lamina IX, were made under visual control with a Multiclamp 700B (Axon Instruments), using glass microelectrodes (3–6 MΩ) filled with the following (in mM): K Gluconate 120, KCl 20, MgCl_2_ 0.1, EGTA 1, HEPES 10, CaCl_2_ 0.1, GTP 0.1, cAMPc 0.2, Leupeptine 0.1, D-mannitol 77, Na_2_-ATP 3, with a pH of 7.3. All experiments were conducted at room temperature (23°C). Voltage- or current- clamp recordings were obtained using a Multiclamp 700B amplifier (Axon Instruments, CA, United States). Data acquisition and analysis were performed using Axograph software. All intracellular recordings were made in high cation aCSF containing 7.5 mM CaCl_2_ and 8 mM MgSO_4_ in order to decrease polysynaptic transmission. Throughout recordings, GABAergic and glycinergic inputs were blocked with gabazine and strychnine (1 μM each), respectively.

### Motoneuron Intrinsic Properties

Input membrane resistance was determined from voltage-current curves obtained from MNs held at −60 mV. After-hyperpolarization (AHP) parameters were computed from single action potential (AP) elicited by injecting a brief depolarizing current pulse (7 ms, 0.25 nA). MN firing behavior was studied using series of depolarizing current pulse injections with increasing amplitude. Then, the instantaneous frequency of firing (*f*-I) was calculated and fitted with linear functions to compute the slope of the linear part (first three points) of the function. MNs were held at −60 mV by bias current application ranging from −500 pA to + 150 pA in current clamp mode.

### Synaptic Responses and Plasticity

Synaptic responses and activity-dependent synaptic plasticity were evoked as previously described (Lenschow et al., 2016). A bipolar stimulating tungsten electrode was placed in the ventrolateral quadrant of the spinal cord slice to stimulate axons of the VLF. Excitatory postsynaptic currents (EPSCs) were evoked in MNs held at −60 mV in voltage-clamp mode by monophasic constant current pulses (100 μs) delivered through a stimulus isolation unit (ISO-flex, AMPI) to a bipolar stimulating tungsten electrode (Microprobes, tip separation 75 μm, stimulation intensities ranging from 10 to 60 μA). After a stable period of 10 min paired-pulse VLF stimulation (50 ms interval applied at 0.03 Hz), a 50 Hz high frequency stimulation (HFS) was applied for 2 s to VLF axons (VLF-HFS). During VLF-HFS, the intensity of the VLF stimulation was doubled compared to the baseline condition for eliciting EPSCs, and MNs were held in current-clamp mode to allow normal depolarization and firing. VLF-EPSC amplitudes were expressed as values normalized to mean control pre-HFS VLF-EPSC amplitude values. MNs were categorized into short-term depression (STD)-expressing motoneurons when VLF-EPSC amplitude exceeded 80% of the pre-HFS baseline control value 500 s after HFS and into long-term depression (LTD)-expressing motoneurons when depression lasted throughout the recording period (more than 30 min).

### Pharmacology

All drugs were prepared as stock solutions, aliquoted and frozen until use. The following pharmacological agents from Abcam (Cambridge, United Kingdom) were used: gabazine (GABA_*A*_ antagonist; 1 μM), a group I/II metabotropic glutamate receptor (mGluR) antagonist MCPG (methylene cyclopropyl glycine; 200 μM); a group I mGluR agonist DHPG (3,5-dihydroxy- phenylglycine; 5 μM); a group II mGluR agonist LY354740 [(1S,2S,5R,6S)-2-aminobicyclo[3.1.0]hexane-2,6-dicarboxylic acid; 0.5 μM] and a group III mGluR agonist L-AP4 (l-2-amino-4-phosphonobutylate; 1 μM). The concentration of the different mGluR agonists used in the present study was determined by performing dose-response curves to reach similar inhibitory effects on the glutamatergic synaptic transmission (data not shown, see section “RESULTS”). The glutamate transporter inhibitor TBOA (DL-threo-benzyloxyaspartic acid; 5 μM) was purchased from Tocris (Bristol, United Kingdom), and the glycine receptor antagonist strychnine (1 μM) from Sigma-Aldrich (St. Louis, MO, United States).

### Electromyographic Recordings and Swimming Monitoring

Animals were anesthetized with isoflurane and implanted with bipolar electrodes (coated nichrome wire, 50 μm; A-M Systems, Carlsborg, WA, United States) directly pinned into the *Gastrocnemius* or *Tibialis Anterior* muscles under binocular control. Due to the small size of the pups, only one muscle per hindlimb was recorded (i.e., one *Gastrocnemius* or one *Tibialis Anterior*). Animals were left to recover from anesthesia until spontaneous movement production and then placed into a tank filled with warm (ca. 35°C) water. Spontaneous swimming activity was recorded during approximately 10 s. Electromyographic signals were amplified (x 5000) using custom-made amplifiers, and filtered at 2 kHz. Data acquisition and analysis were performed using Axograph software. After three sessions of control swimming activity spaced 5 min apart, mice were randomly assigned to the following four treatment groups: vehicle (aCSF), DHPG (20 mg/kg), LY354740 (0.5 mg/kg), or L-AP4 (10 mg/kg). These concentrations were determined by an extrapolation from the concentrations used for intracellular recordings by a simple conversion based on mouse pup weight. The DHPG concentration has to be increased due to an absence of effects *in vivo*, while the LY354741 concentration was reduced due to a complete blockage of the swimming activity in pups (data not shown). Solutions were injected subcutaneously using an Hamilton syringe (Hamilton, Reno, NV, United States) at a volume of 0.3 μL for P3 and 1 μL for P8-P10 animals. Swimming activity was recorded 5, 10, 20 and 30 min after the injection. Between each session, mouse pups were placed on a heating pad.

### Reverse Transcription and Real-Time Quantitative Polymerase Chain Reaction

The ventral lumbar part of the spinal cord or individual L2 and L5 ventral segments were isolated from isoflurane-anesthetized mouse pups and immediately frozen at −80°C until use. During all procedures, care was taken to avoid RNA degradation. Total RNA was extracted from samples using the *RNA NucleoSpin*^®^ *RNA Plus extraction kit* (Macherey-Nagel, Hoerdt, France). RNA quality and quantity were analyzed using Agilent RNA 2200 Screen Tape system (Agilent Technologies, Les Ulis, France) and DS-11 spectrometer (DeNovix, Wilmington, NC, United States) respectively.

To perform RT-qPCR from identified MNs innervating the *Gastrocnemius* or *Tibialis Anterior* muscles, MNs were backfilled with cholera toxin ß-subunit (CTB) conjugated with Alexa Fluor 594 or Alexa Fluor 488 (C34775 and C34777, respectively, Thermo Scientific, Waltham, MA, United States). Both muscles of both hindlimbs were injected in anesthetized P0 mice. After 12 h of migration, the lumbar spinal cord was dissected out from anesthetized animals, cryosectioned into longitudinal sections (20 μm) and collected on polyethylene-naphthalate membrane RNase-free microscope slides. Immediately after dehydration in successive baths of ethanol at increasing concentrations, labeled MN somas were dissected at 63x magnification using a PALM laser microdissection and capture system (P.A.L.M. Microlaser Technologies AG, Bernried, Germany). *Gastrocnemius* or *Tibialis* MNs were collected in separate tubes with adhesive caps for no longer than 30 min per slide to limit RNA degradation. The collected material was then treated with lysis buffer and stored at −80°C until RNA extraction. In the case of laser capture microdissection (LCM) samples, the *ReliaPrep*™ *RNA Cell Miniprep System* from Promega (Promega, La Farlede, France) was used for RNA extraction. The RNA integrity and quantity were checked by the Bioanalyser 2100 (Agilent Technologies, Massy, France) and the Nanodrop 1000 (Thermo Scientific, Waltham, MA, United States), respectively.

For all the samples used, total RNA was reverse-transcribed to cDNA with *GoScript*™ *Reverse Transcription Kit* (Promega) by standard protocols. Samples were distributed in duplicate using the Eppendorf epMotion 5073 automated pipetting. PCR was performed with the *GoTaq Master Mix* from Promega and the CFX384 real-time PCR detection system (Bio-Rad, Marnes-la-Coquette, France). Cycling parameters for the qPCR reaction included a 3 min hot start followed by 40 cycles of denaturation at 90°C for 10 s, annealing at 60°C for 30 s, and elongation at 72°C for 30 s. Sdha (succinate dehydrogenase) and actine ß (Actß) were used as internal controls. Two technical replicates were run for each RT-qPCR experiments and relative expression levels were calculated using the 2^–ΔΔCt^ method after normalization to the Sdha housekeeping gene. The primers sequences used (Table 1) were provided by Sigma Genosys (The Woodlands, TX, United States).

**TABLE 1:** List of primers used for Reverse transcription and real-time quantitative polymerase chain reaction (RTq-PCR) analysis.

Gene	Sequence (5′ to 3′)	Accession number
Sdha	GGAACACTCCAAAAACAGACCT	NM_023281.1
	CCACCACTGGGTATTGAGTAGAA	
Actß	GTGGGAATGGGTCAGAAGGA	NM_007393.5
	TACATGGCTGGGGTGTTGAA	
Grm1	ACAAAAGCGGAATGGTACGA	NM_016976.3
	AGGCTCTGCAGGTAAACTCA	
Grm2	TCAATGCCGTGTATGCCATG	NM_001160353.1
	TGTAGCGGCCAATACCATCT	
Grm3	GAGTCATTGGCGGTTCGTAC	NM_181850.2
	TCCCTGTCTCCCCATAGTCA	
Grm4	TGACCGATACTTCTCCAGCC	NM_001013385.2
	CCTTCCCCTCCTGTTCGTAG	
Grm5	TATGTCTCAGCTGTGCACAC	NM_001143834.1
	CGAACTGTCATGCCTTCACA	
Grm7	GTTGGAGTGATTGGGGCTTC	NM_177328.3
	TGGAAGGAATCAGGTGGGAC	

### Statistical Analysis

Statistical analysis was conducted on raw data using GraphPad Prism software. The data were checked for normal distribution. Wilcoxon matched pairs or Mann–Whitney tests were used to compare two series of data. Kruskal–Wallis one-way analysis of variance (ANOVA) was carried out to test for significant effects between the different drugs for unpaired observations. Two-way or RM two-way ANOVAs followed by Sidak’s multiple comparison tests were performed to evaluate drug and age effects. Chi-square tests were used to compare motoneuron proportions displaying STD, LTD or no change in the different conditions tested. In the text and figures, all data are expressed as means ± SEM. Statistical significance level was set at *p* < 0.05.

## Results

### Metabotropic Glutamate Receptors at Ventrolateral Funiculus-Motoneuron Synapses

**FIGURE 1:**
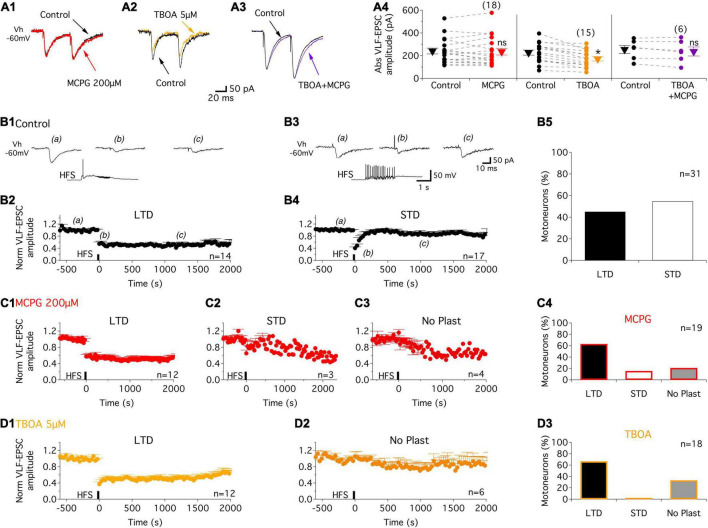
Increased glutamatergic release activates mGluR receptors and contributes to activity-dependent synaptic plasticity (ADSP) expressed at VLF-MN synapses. **(A)** Representative traces of EPSCs triggered by paired stimulations (50 ms interval) applied to VLF axons in lumbar MNs held at –60 mV (Vh –60 mV) in P1-P3 mice in control condition (black traces) and in the presence of the broad-spectrum antagonist, MCPG, (red trace, **A1**), the glutamate transporter inhibitor, TBOA, (orange trace, **A2**) and MCPG + TBOA (purple trace, **A3**). **(A4)** Note that the depressing effect of TBOA on VLF-EPSC amplitudes was suppressed in the presence of MCPG. Scatter plots with connecting lines (dashed) of the absolute EPSC amplitude values (Abs VLF-EPSC) in the different experimental conditions. ns: non-significantly different, Wilcoxon test, *p* = 0.6 for MCPG and *p* = 0.1 for TBOA + MCPG. *Significantly different, Wilcoxon test, *p* < 0.0001 for TBOA. The triangles correspond to means ± sem of the population tested in each condition and numbers in parentheses to the number of MNs tested. **(B)** Representative experiment illustrating VLF-HFS (50 Hz, 2 s)-induced LTD **(B1,B2)** and STD **(B3,B4)** in control condition in P1-P3 mice. Sample traces of VLF-EPSC before (a), at different time points after (b and c) and during VLF-HFS application (lower trace) in an LTD-expressing MN **(B1)** and a STD-expressing MN **(B3)**. **(B2,B4)** Pooled data showing average time courses of normalized VLF-EPSC amplitudes in MN expressing LTD **(B2)** or STD **(B4)** in control condition in P1-P3 mice. **(B5)** Percentage of MNs expressing the different types of ADSP. **(C)** Pooled data for LTD **(C1)** and STD **(C2)** time courses or an absence of synaptic plasticity (No Plast, **C3**) after VLF-HFS in the presence of MCPG. **(C4)** Histogram of MN repartition according of ADSP expressed following VLF-HFS. **(D)** Same as in **(C)** but in the presence of TBOA. Note the absence of STD induction. *n*, number of MNs.

In the baseline control condition, paired-pulse stimulation (50 ms interval) applied to the VLF in slices triggered excitatory postsynaptic currents (VLF-EPSCs) in lumbar MNs held at −60 mV (Figure 1A). As previously shown by our group (Lenschow et al., 2016), VLF-EPSCs exhibit paired-pulse facilitation (PPF) with this stimulation paradigm (Figures 1A1–A3). In 1 week old rat pups, MCPG, a broad spectrum mGluR antagonist has been shown to block the late component of dorsal root response in lumbar MNs but failed to affect VLF-induced responses (Arvanian et al., 2005). We observed a similar lack of effect of 200 μM MCPG on both VLF-EPSC amplitude (Figures 1A1,A4) and the PPF ratio (VLF-EPSC2/VLF-EPSC1 = 1.3 ± 0.3 in control condition and 1.3 ± 0.3, *n* = 18 in the presence of MCPG, *p* = 0.5, Wilcoxon matched-pairs test) in P1-P3 mice. At excitatory glutamatergic synapses, mGluRs, in contrast to NMDA and AMPA receptors that are enriched in the postsynaptic density, are localized perisynaptically and have been shown to be activated when high concentrations of glutamate are reached in the synaptic cleft (Scheefhals and MacGillavry, 2018). We therefore examined the effects of enhancing endogenous glutamate level on VLF-EPSC amplitude with the glutamate transporter inhibitor TBOA. The bath-application of 5 μM TBOA on spinal cord slices significantly decreased the VLF-EPSC amplitudes (Figures 1A2,A4). However, this depressive effect was counteracted when MCPG was added to the TBOA-containing aCSF (Figures 1A3,A4). These results thus indicate that increasing the endogenous glutamate concentration not only activates mGluRs, but also that the latter might be acting as inhibitory autoreceptors at VLF-MN synapses.

### Modulation of Activity-Dependent Synaptic Plasticity at Ventrolateral Funiculus-Motoneuron Synapses by Metabotropic Glutamate Receptors

It has been previously shown that the activation of extrasynaptic mGluRs by high synaptic concentrations of glutamate that spill over the synaptic terminals is a major determinant of synaptic plasticity expressed in supraspinal structures (Gonçalves-Ribeiro et al., 2019; Valtcheva and Venance, 2019). We thus investigated whether the activation of mGluRs at VLF-MN synapses during VLF high-frequency contributes to the ADSP expression at these synapses. As previously reported (Lenschow et al., 2016), a 2 s 50 Hz stimulation applied to VLF axons (VLF-HFS), that mimic the discharge of reticulospinal neurons during locomotion (Matsuyama and Drew, 2000a,b), leads to a long (more than 30 s; Figures 1B1,B2) or a short-term (Figures 1B3,B4) depression (LTD, STD respectively) of VLF-EPSC amplitudes at VLF-MN synapses in control conditions in P1-P3 mice. LTD and STD were almost equally distributed amongst the 31 recorded lumbar MNs (Figures 1B5). Of 19 MNs then exposed to the mGluR antagonist MCPG, LTD (Figures 1C1,C4) and STD (Figures 1C2,C4) were still expressed in 12 and 3 MNs, respectively, whereas in the remaining 4 MNs, VLF-HFS failed to trigger any changes in VLF-EPSC amplitudes (No plasticity, Figures 1C3,C4).

To amplify the glutamate release induced by HFS and to further activate potential perisynaptic mGluRs at VLF-MN synapses, VLF-HFS was applied in the presence of TBOA. As shown in Figure 1D, in these experimental conditions, it was still possible to induce LTD (Figures 1D1,D3) of VLF-EPSC amplitudes but the expression of STD was completely blocked in lumbar MNs (Figures 1D2,D3). The magnitudes of depression of VLF-EPSC amplitudes observed during LTD or STD in the presence of MCPG were not significantly different from those in the control condition: LTD: control −45 ± 6%, *n* = 14, MCPG −42.4 ± 4%, *n* = 12 and STD: control −59.3 ± 4%, *n* = 17 and MCPG −34.6 ± 8%, *n* = 3 (Table 2). In contrast, a significant increase in LTD magnitude was observed in the presence of TBOA (compare Figures 1B2,D1): control −45 ± 6%, *n* = 14, TBOA −62.8 ± 4%, *n* = 12, Mann-Whitney test, *p* = 0.04). Altogether these data indicate that mGluRs are activated during VLF-HFS and contribute to the expression of ADSP at VLF-MN synapses.

**TABLE 2:** Results of the repeated measure two-way analysis of variance (ANOVA) performed to test the magnitude of depression reached during long term depression (LTD) and short-term depression (STD) (Plasticity) in the presence of methylene cyclopropyl glycine (MCPG) (Drug).

				MCPG			
	Fig nb	Interaction		Drug		Plasticity	
		F	p	F	p	F	p
Magnitude of depression	1	(1,42) 3	0.1	(1,42) 0.2	0.6	(1,42) 4	0.05

*The numbers into brackets correspond to the degrees of freedom. Fig nb: number of the corresponding Figure.*

**FIGURE 2:**
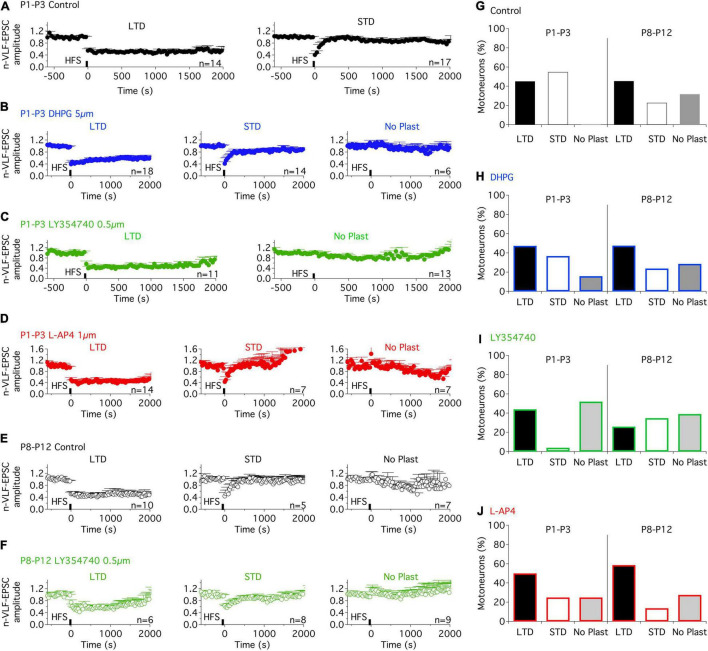
Metabotropic glutamate receptor (mGluR) agonists differentially modulate the ADSP expressed at VLF-MN synapses during development. **(A)** Pooled data average time courses of VLF-HFS-induced LTD (left panel) or STD (right panel) in lumbar MNs of P1-P3 mice in control condition. **(B)** Same layout as in **(A)** (note, with addition of the time course in the absence of plasticity, right) in the presence of the mGluRI agonist, DHPG. **(C)** Plasticity time courses in the presence of the mGluRII agonist, LY354740 in P1-P3 MNs. Note the absence of STD in the presence of LY354740. **(D)** LTD, STD and No Plast were expressed in the presence of the mGluRIII agonist, L-AP4 in P1-P3 MNs. **(E)** Pooled data average time courses of VLF-HFS-induced LTD (left panel), STD (middle panel) or No Plasticity (right panel) in lumbar MNs of P8-P12 mice in control condition. **(F)** Same layout as in E in the presence of the mGluRII agonist, LY354740. **(G–J)** Percentages of MNs expressing each type of synaptic plasticity profile in control condition **(G)**, in the presence of DHPG **(H)**, LY354740 **(I)** and L-AP4 **(J)** for the two developmental stages tested. Note that the ADSP expression was similar in control condition and in the presence of the mGluR agonists in P8-P12 MNs. The distribution of the different ADSP profiles expressed in the presence of mGluR agonists was found significantly different in P1-P3 MNs but not in P8-P12 MNs in the presence of the mGluR agonists (Chi square test, *p* < 0.0001 and *p* = 0.44, respectively).

To assess the separate roles of the three different groups of mGluRs in the modulation of ADSP at VLF-MN synapses, we tested the impact of exposure to specific mGluR agonists, DHPG, LY354740, and L-AP4 for mGluRI, mGlurII, and mGluRIII, respectively (see section “DISCUSSION”). The agonists were applied to the slices throughout recording periods. 10 min after the onset of each agonist’s superfusion, a 10 min period of stable low frequency (0.03 Hz) VLF stimulation was applied prior to VLF-HFS application. In P1-P3 mice, as in the control condition (Figures 2A,G), in the presence of DHPG, VLF-HFS elicited both LTD and STD (Figures 2B,H) but also failed to induce changes in VLF-EPSC amplitudes in some of the lumbar MNs tested (Figures 2B,H). Similar diverse effects were observed in the presence of the mGluRIII agonist, L-AP4 (Figures 2D–J). LTD was expressed in 44% of the MNs tested, STD in 36.8% and No Plast in 15.8% in the presence of DHPG (Figure 2H), while with L-AP4, LTD-expressing MNs represented 50% of the neurons tested, STD-expressing MNs 25% and No Plast-expressing ones 25% (Figure 2J). When mGluRsII were activated with LY354740, LTD was observed in 44% of the MNs recorded (Figures 2C,I), but only one of the 25 tested MNs presented an STD (4%, Figure 2I) after the VLF-HFS. The 52% remaining neurons recorded in the presence of LY354740 exhibited no synaptic plasticity (Figures 2C,I), indicating that the mGluRII agonist was causing an almost complete and selective blockade of STD expression at VLF-MN synapses.

To assess for developmental changes, the same experiments were conducted on lumbar MNs recorded from P8-P10 mice. At this maturational stage, in control conditions, VLF-HFS again led to either LTD, STD or no plasticity (Figures 2E, G) as previously reported (Lenschow et al., 2016). When applied in the presence of each of the three mGluR agonists tested, VLF-HFS also elicited one of the same three possible responses - i.e., LTD, STD and No Plast- at VLF-MN synapses, regardless of the compound tested (Figures 2F,H,I,J). There were also no significant differences between the three different agonists in terms of the number of MNs expressing a particular response (Figures 2H,I,J; Chi square test, *p* = 0.45). Moreover, the magnitudes of depression of VLF-EPSC amplitudes attained during LTD or STD were not significantly different in control condition and in the presence of each of the three mGluR agonists (Table 3). Altogether these data therefore suggest that in contrast to P1-P3 MNs, mGluR activation does not influence the expression of ADSP induced by a HFS at VLF-MN synapses of P8-P10 mice.

**TABLE 3:** Magnitude in percentage of the depression reached during long term depression (LTD) or short-term depression (STD) in control condition and in the presence of the three different mGluR agonists for the two different developmental stages tested.

		P1-P3	P8-P12
**LTD**			
	Control	−45 ± 6% ν = 14	−49 ± 6% ν = 10
	DHPG	−60 ± 5% ν = 18	−43 ± 8% ν = 10
	LY354740	−43 ± 11% ν = 11	−25 ± 7% ν = 6
	L-AP4	50 ± 9% *n* = 14	−47 ± 4% ν = 17

**STD**			
	Control	−59 ± 4% ν = 17	−56 ± 12% ν = 5
	DHPG	−59 ± 6% ν = 14	−46 ± 9% ν = 5
	LY354740	NA	−36 ± 5% ν = 8
	L-AP4	−57 ± 7% ν = 7	−62 ± 1% ν = 4

*NA, non-applicable; STD was abolished in the presence of LY354740 in P1-P3 mice. n, number of MNs tested in each condition. No significant difference were observed, two way ANOVA, for LTD, interaction, F(_53,92_) = 1, p = 0.4; age, F(_3,92_) = 2, p = 0.2; drug, F(_1,92_) = 2.5, p = 0.1; for STD: interaction, F(_2,46_) = 0.6, p = 0.6; age, F(_2,46_) = 0.4, p = 0.7; drug, F(_1,46_) = 0.2, p = 0.6; followed by Sidak’s post hoc tests.*

### Effects of Metabotropic Glutamate Receptor Activation on the Synaptic Transmission at Ventrolateral Funiculus-Motoneurons Synapses

**FIGURE 3:**
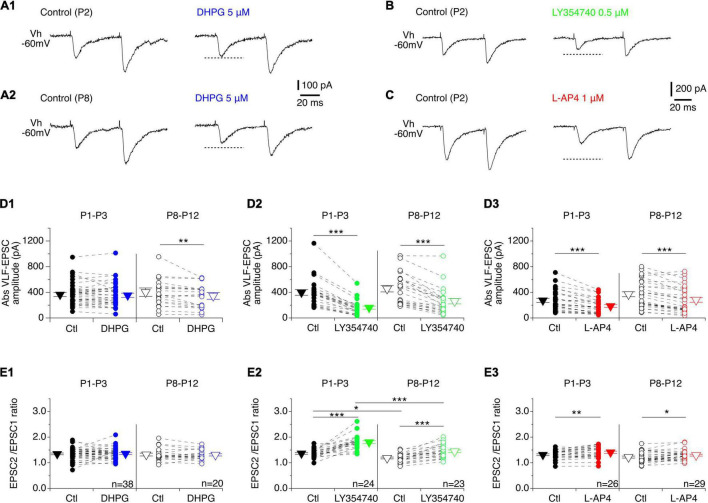
Effects of mGluR agonists on VLF-MN synaptic transmission. **(A)** Representative traces of EPSCs induced by paired stimulations (50 ms interval) applied to VLF axons in lumbar MNs held at –60 mV (Vh –60 mV) in a P2 **(A1)** and a P8 **(A2)** mouse in control condition (left panels) and in the presence of DHPG (right panels). Note the absence of effect of DHPG in P2 MNs. **(B)** Sample traces recorded from a P2 lumbar MN during paired-pulse VLF stimulations showing the decrease in EPSC amplitude observed in the presence of LY354740 compared to control condition. **(C)** Same as in B for a P2 MN recorded in control condition and in the presence of L-AP4. **(D)** Scatter plots with connecting lines (dashed) of absolute VLF-EPSC amplitudes recorded in P1-P3 (filled circles) and P8-P12 MNs (open circles) before (black circles) and after exposure to DHPG (blue circles, **D1**), LY354740 (green circles, **D2**) or L-AP4 (red circles, **D3**). **(E)** Same data presentation as in **(D)** for mean EPSC2/EPSC1 ratios. DHPG was ineffective in affecting the EPSC2/EPSC1 ratio in both P1-P3 and P8-P12 MNs, while LY354740 and L-AP4 significantly increased it at both developmental stages tested. The triangles correspond to means ± sem of the population tested in each condition. Two-way RM ANOVA followed by Sidak’s *post hoc* tests, *p < 0.05; **p < 0.01 and ***p < 0.001 significantly different.

In a next step, we tested whether mGluR activation was accompanied by a modulation of the synaptic inputs received by MNs. Under baseline conditions of VLF stimulation that resulted in PPF, DHPG had no effect on VLF-EPSC amplitudes in P1-P3 MNs (Figures 3A1,D1), but caused a significant decrease in this parameter in P8-P12 MNs (Figures 3A2,D1 and Table 4, VLF-EPSC ampl). The presence of the mGluRI agonist did not significantly modify the PPF ratio at both developmental stages tested (Figures 3A,E1 and Table 4, EPSC2/EPSC1 ratio). In contrast, LY354740 (Figure 3B) and L-AP4 (Figure 3C) induced a substantial decrease in VLF-EPSC amplitude in both P1-P3 and P8-P10 lumbar MNs (Figures 3D2,D3 and Table 4) that was consistently accompanied by an elevation in the PPF ratio (Figures 3E2,E3 and Table 4), suggesting a presynaptic locus of mGluR II and mGluR III modulation.

**TABLE 4:** Results of the repeated measure two-way analysis of variances (ANOVAs) performed to test the effects of mouse age (age, P1-P3 vs. P8-P12) and metabotropic glutamate receptor (mGluR) agonists (drug) on the different electrophysiological parameters tested.

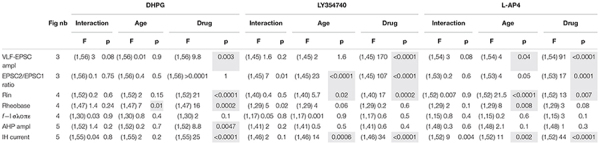

*The numbers into brackets correspond to the degrees of freedom. Ampl, amplitude; Rin, input membrane resistance; f-I slope, slope of the frequency-current relationship. Fig nb, number of the corresponding Figure. Shaded cells, p < 0.05 significantly different.*

### Metabotropic Glutamate Receptor-Induced Modulation of Motoneuron Intrinsic Membrane Properties

**FIGURE 4:**
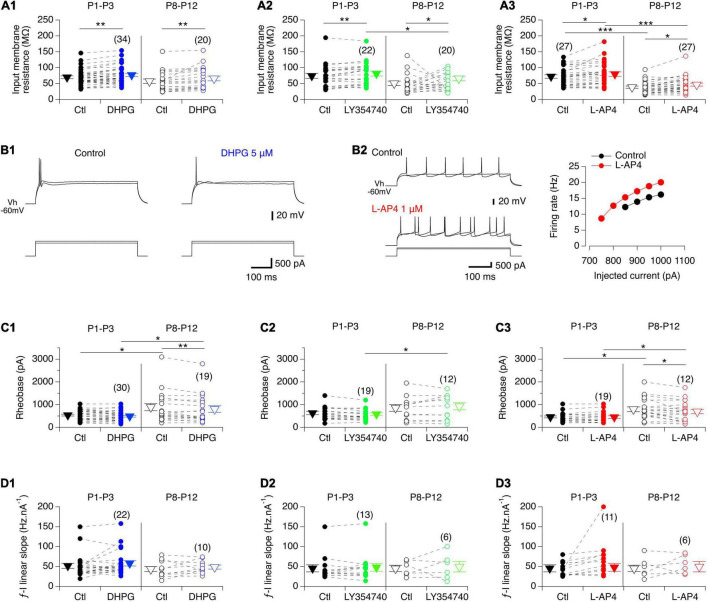
Effects of the three mGluR agonists on membrane input resistance and firing properties of lumbar MNs. **(A)** Scatter plots with connecting lines (dashed) of mean membrane input resistance of P1-P3 (filled circles) and P8-P12 MNs (open circles) before (black circles) and after exposure to DHPG (blue circles, **A1**), LY354740 (green circles, **A2**) or L-AP4 (red circles, **A3**). All three compounds significantly increased the membrane input resistance of MNs regardless of the developmental stage tested. **(B)** Representative traces of membrane potential responses to injected depolarizing pulses in the absence and presence of DHPG in a P3 MN **(B1)** or of L-AP4 in a P8 MN (left panel in **B2**; right panel: corresponding plot of the mean spike frequency as a function of the injected current). **(C)** Same data representations as in A for the rheobase. DHPG and L-AP4 significantly decreased the rheobase value in MNs at P8-P12. **(D)** Same data representations as in A for the slope of the *f*-I relationships showing that none of the three mGluR agonists tested altered this parameter. The triangles correspond to means ± sem of the population tested in each condition and numbers in parentheses to the number of MNs tested. Two-way RM ANOVA followed by Sidak’s *post hoc* tests, *p < 0.05 and **p < 0.01 significantly different.

As previously described (Vinay et al., 2000; Smith and Brownstone, 2020), we observed that the input membrane resistance of MNs significantly decreased with age: mean 72 ± 3 MΩ, *n* = 86 from 65 P1-P3 pups versus 48 ± 3 MΩ, *n* = 67 from 59 P8-P12 mice, Mann-Whitney test, *p* < 0.0001. Regardless of age, the three mGluR agonists had no significant effect on the resting membrane potential of MNs when bath-applied in current clamp mode but significantly increased their input membrane resistance (Figure 4A and Table 4, Rin). We then sought whether mGluR activation changed the instantaneous frequency-current (*f*-I) relationship in lumbar MNs by applying depolarizing current pulses injected in series (Figure 4B). In contrast to previous findings (Vinay et al., 2000; Smith and Brownstone, 2020), we observed that the rheobase computed in control condition was significantly increased in P8-P10 MNs compared to P1-P3 MNs (P1-P3: 543 ± 30 pA, *n* = 68 and P8-P12: 866 ± 80 pA, *n* = 49; Mann-Whitney test, *p* = 0.0006). During this protocol, two different types of responses were observed when spike threshold was reached: 22 out of 68 (32%) P1-P3 MNs and 29 out of 51 (43%) P8-P12 MNs exhibited only a solitary spike at the beginning of the depolarizing pulse (Figure 4B1), whereas in the remaining cells, tonic impulse firing occurred throughout the depolarization (Figure 4B2). Neither firing pattern was modified in the presence of any of the three mGluR agonists tested (examples shown for DHPG and L-AP4 in Figures 4B1,B2, respectively; data not shown for LY354740). Unexpectedly, we observed no significant influence of the three mGluR agonists on the rheobase (Figure 4C, and Table 4, Rheobase) at P1-P3 nor on the slope of the *f*-I relationships in the two age ranges examined (Figure 4D and Table 4, *f*-I slope). In P8-P10 MNs, however, DHPG and L-AP4 (Figures 4C1,C3), but not LY354740 (Figure 4C2) significantly decreased the MN rheobase (Table 4, Rheobase).

**FIGURE 5:**
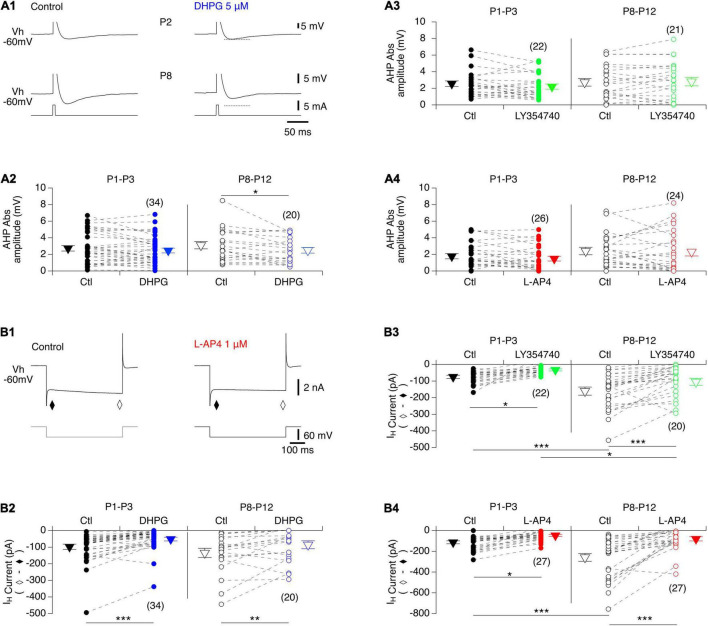
Actions of mGluR agonists on AHP and I_*H*_ current in lumbar MNs. **(A)** Representative AHPs expressed after an action potential induced by a brief current pulse injected into a P2 (**A1**, top panel) and a P8 (**A1**, bottom panel) MN in control condition (**A1**, left) and in the presence of DHGP (**A1**, right) held at –60 mV (Vh –60 mV). The action potentials have been truncated for better visualization of the AHPs. Dashed lines correspond to the value of AHP amplitude in control. **(A2–A4)** Scatter plots with connecting lines (dashed) of absolute mean AHP amplitudes computed in P1-P3 (filled circles) and P8-P10 MNs (open circles) before (black circles) and after exposure to DHPG (blue circles, **A2**), LY354740 (green circles, **A3**) or L-AP4 (red circles, **A4**). Note that AHP amplitudes were significantly decreased only in the presence of DHPG in P8-P12 MNs. **(B)** Sample membrane current traces obtained in response to a voltage step of –60 mV in a MN held at –60 mV (Vh –60 mV) in voltage clamp conditions before (control) and after exposure to L-AP4 on the slice **(B1)**. The I_*H*_ current was computed by subtracting the instantaneous current (τ) from the steady state (♢). Regardless of age, all three mGluR agonists caused a significant blockage of the I_*H*_ current. **(B2–B4)** Scatter plots with connecting lines (dashed) of the I_*H*_ current in P1-P3 (filled circles) and P8-P10 MNs (open circles) before (black circles) and after exposure to DHPG (blue circles, **B2**), LY354740 (green circles, **B3**) or L-AP4 (red circles, **B4**). The triangles correspond to means ± sem of the population tested in each condition and numbers in parentheses to the number of MNs tested. Two-way RM ANOVA followed by Sidak’s *post hoc* tests, *p < 0.05; **p < 0.01 and ***p < 0.001 significantly different.

We then examined whether the after-spike hyperpolarization (AHP), a major target of neuromodulators in MNs, was modified in the presence of the mGluR agonists. In control conditions, the AHP amplitude values were found not to be significantly different between P1-P3 and P8-P12 MNs (2.4 ± 0.2 mV, *n* = 82 and 2.7 ± 0.2 mV, *n* = 65, respectively; Mann-Whitney test, *p* = 0.3). As illustrated in Figures 5A1,A2, a significant decrease in AHP amplitudes was found only in the presence of DHPG in P8-P10 MNs, whereas for the other two mGluR agonists tested, no significant effects were observed in either age range (Figures 5A3,A4 and Table 4, AHP ampl). This finding is therefore consistent with the lack of effect of the agonists on the *f*-I relationships of MNs, which have been shown to be closely correlated with AHP amplitudes (Fulton and Walton, 1985).

To complete this analysis of the cellular basis of mGluR modulation in lumbar MNs, we examined the impact of the three mGluR agonists on the hyperpolarization-activated mixed cation current I_*H*_, another important cellular target of neuromodulatory systems in the spinal cord (Deardorff et al., 2021). For this purpose, voltage steps of -60 mV from a holding membrane potential of -60 mV were applied and the instantaneous current evoked was measured immediately after the capacitive transient (filled diamond in Figure 5B1) and the steady-state current at the end of the hyperpolarizing pulse (open diamond in Figure 5B1) in control condition and in the presence of each mGluR agonist. As previously established, the difference between the steady state and the instantaneous current represents the I_*H*_ current (Bertrand and Cazalets, 1998; Kjaerulff and Kiehn, 2001; Tartas et al., 2010). The amplitude of I_*H*_ computed in P8-P12 MNs (−192 ± 19 pA, *n* = 67) was found to be significantly higher than in P1-P3 MNs (−96 ± 8 pA, *n* = 83, Mann-Whitney test, *p* < 0.0001, Figures 5B2–B4), and all three mGluR agonists significantly decreased I_*H*_ amplitude regardless of age (Figure 5B and Table 4).

Together these results show that mGluR modulation in lumbar MNs is complex, with specific effects being associated with a specific mGluR subtype and developmentally regulated.

### Metabotropic Glutamate Receptor Gene Expression in the Ventral Spinal Cord

**FIGURE 6:**
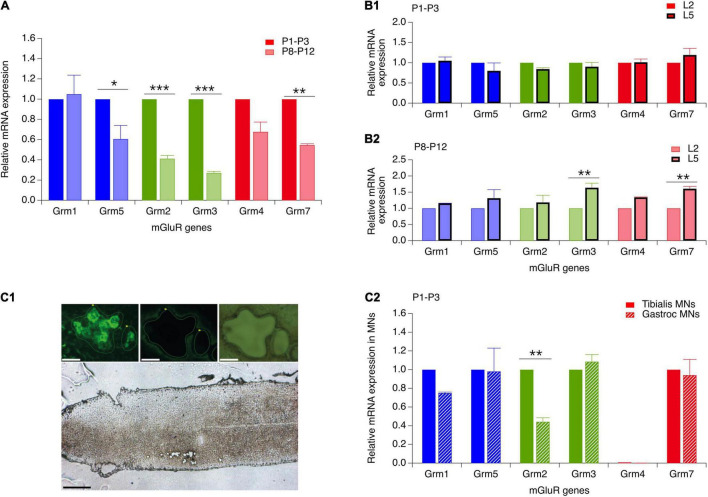
Reverse transcription and real-time quantitative polymerase chain reaction (RT-qPCR) analysis of the expression of mGluR genes in the developing ventral spinal cord and in flexor and extensor MNs. **(A)** mRNA expression of genes coding for mGluR1-5,7 in the ventral spinal cord of P8-P12 mice (hatched bars) relative to their respective expression in P1-P3 animals (solid bars). **(B)** mGluR gene expression in the ventral L5 spinal segment (thick stroke bars) relative to their respective expression in the L2 segment (thin stroke bars) in P1-P3 **(B1)** and P8-P12 mice **(B2)**. **(C) (C1)** Lower image: longitudinal section of a lumbar spinal cord of a P3 mouse after the laser microdissection of motoneurons. Scale bar: 300 μm. Upper images: magnification of motoneurons from the *Gastrocnemius* muscle that were retrogradely labeled with CTB before (left) and after (middle and right panel) laser microdissection. Scale bars: 30 μm. **(C2)** mGluR gene expression in P1-P3 *Gastrocnemium* motoneurons (dashed bars) relative to the expression of the same genes in P1-P3 *Tibialis* motoneurons (solid bars). Two-way RM ANOVA followed by Sidak’s *post hoc* tests. *p < 0.05 and **p < 0.01 significantly different.

At present, there are only few data available concerning the developmental expression of the various mGluR genes in the motor spinal cord (Berthele et al., 1999). To further explore these developmental changes, we looked for age-related modifications in expression levels of six mGluR genes (Grm) known to be present in the mouse spinal cord: Grm1, 2, 3, 4, 5, and 7 (Valerio et al., 1997; Berthele et al., 1999) by RT-qPCR analysis. In the whole lumbar ventral spinal cord of P1-P3 and P8-P12 mice, all 6 mGluR gene subtypes were found to be present in the two age ranges (*n* = 12 mice per age group), although at least four subtypes, mGluR5, mGluR2, mGluR3 and mGluR7, were significantly down-regulated during the second postnatal week (Figure 6A and Table 5, P8-P12 vs P1-P3).

**TABLE 5:** Results of the repeated measure two-way analysis of variances (ANOVAs) performed to test the effects of mouse age (age, P8-P12 vs. P1-P8), spinal segments (lumbar segment 2 and 5, L5 vs. L2; segments) and muscle targeted by motoneurons (MNs) (*Gastrocnemius* muscle versus *Tibialis muscle*; muscle) on the expression of the different metabotropic glutamate receptor (mGluR) genes.

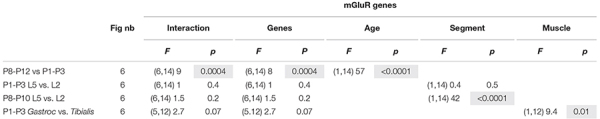

*The numbers into brackets correspond to the degrees of freedom. Fig nb, number of the corresponding Figure. Shaded cells, p < 0.05 significantly different.*

Since the plasticity expressed at VLF-MN synapses is a function of the flexor or extensor function of lumbar MNs (Lenschow et al., 2016) and is modulated by mGluR activation, we next asked whether flexor and extensor MNs exhibit different and specific expression profiles of the mGluR genes. To this end, in a first series of experiments, we compared mRNA levels between the L2 and L5 ventral spinal segments (*n* = 12 animals per age group) which have been shown to contain predominantly flexor and extensor MNs, respectively (Cazalets et al., 1992; Kiehn and Kjaerulff, 1996). In P1-P3 animals, no significant differences in gene expression levels were found between the L2 and L5 segments (Figure 6B1 and Table 5, P1-P3 L5 vs L2). However, in P8-P12 mice, Grm3 and Grm7 were significantly more expressed in L5 compared to the L2 segment (Figure 6B2 and Table 5, P8-P12 L5 vs. L2).

In a next step, we quantified and compared mGluR gene expression levels in laser-microdissected MNs that had been retrogradely labeled from the ankle extensor muscle *Gastrocnemius* or from the ankle flexor *Tibialis Anterior* muscle (Figure 6C1). It should be noted that this analysis was restricted to MNs in P1 animals only, since *Gastrocnemius* MNs have been shown to split into LTD-MNs or No Plast-MNs after VLF-HFS in P8-P12 MNs and could therefore not be considered as an homogenous population in terms of ADSP expression (Lenschow et al., 2016). The mRNA from 1017 *Tibialis* MNs (from 15 P1 mice) and 783 *Gastrocnemius* MNs (from 5 P1 mice) were collected and purified (Rin = 8.7 and 7, respectively). With this experimental approach, all the genes tested were detectable in both MN subtypes with the exception of Grm4 and RT-qPCR analysis revealed that the level of Grm2 expression was significantly lower in *Gastrocnemius* MNs compared to *Tibialis* MNs (Figure 6C2 and Table 5, P1-P3 *Gastroc* vs *Tibialis*). This finding therefore indicated that extensor and flexor MNs exhibit slightly, albeit significantly, different mGluR expression profiles.

As the ADSP expressed at VLF-MN synapses is dependent on the flexor or extensor identity of MNs, we sought whether the effects of the mGluR agonists differ between MNs exhibiting different ADSP profiles at VLF-MN synapses after VLF-HFS. We found that the mGluR-mediated modulations of VLF-EPSC amplitude values, EPSC2/EPSC1 ratio, input membrane resistance and AHP amplitude were similar whatever the ADSP expressed by MNs and at the two developmental stages tested (data not shown).

### Impact of the Metabotropic Glutamate Receptor Agonists on Ventrolateral Funiculus-Induced Fictive Locomotion

**FIGURE 7:**
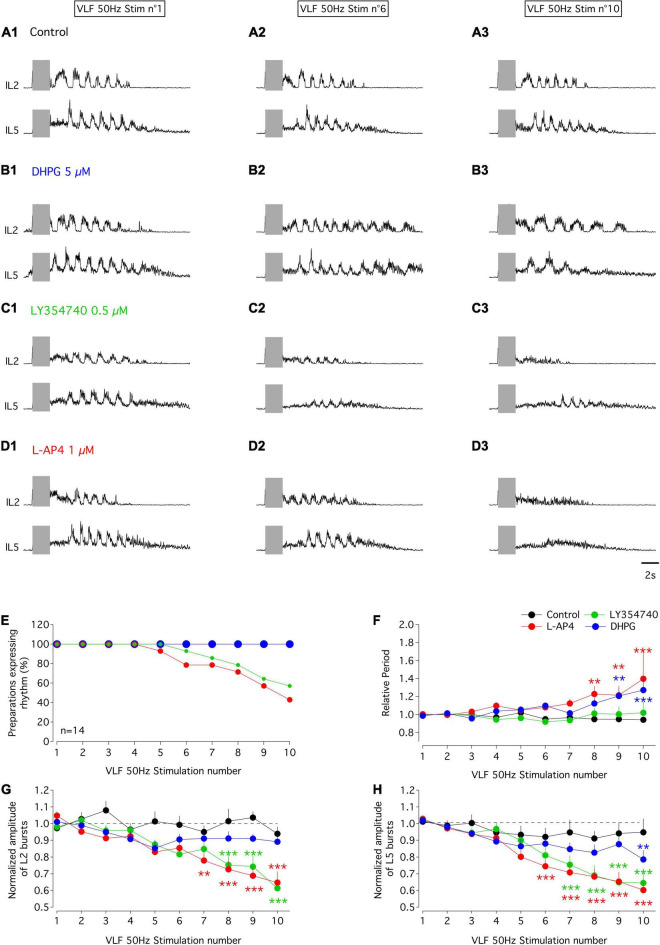
Effects of mGluR agonists on VLF-induced fictive locomotion in the isolated spinal cord *in vitro* of P1-P3 mice. **(A–D)** Representative sample traces of bouts of fictive locomotion induced by the first (left panels), sixth (middle panel) and tenth (right panels) stimulation (gray rectangles) of series of 10 successive stimuli applied to VLF axons (50 Hz, 2 s) every 2 min in a whole spinal cord preparation from a P1 mouse in control condition in aCSF **(A)** or during successive exposure to DHPG **(B)**, LY354740 **(C)** or L-AP4 **(D)**. Each drug application started at the beginning of the stimulation series and was separated by at least an one hour washout period with aCSF. **(E)** Percentages of isolated spinal cord preparations (*n* = 14) expressing locomotor episodes during series of 10 VLF stimulations in control condition and during application of mGluR agonist. Note that the curve obtained in the presence of DHPG is superimposed on that obtained in aCSF. **(F)** Evolution of cycle period of VLF-induced-fictive locomotion rhythm normalized to the mean period computed for the two first stimulations in each series in control condition and during the application of each mGluR agonist. **(G)** Same data representation as in **(F)** for the amplitude of L2 locomotor-related bursts normalized to the mean amplitude value obtained in response to the two first stimulations in the series. **(H)** Same data representation as in **(F)** for the amplitude of L5 locomotor bursts normalized to the mean amplitude value obtained in response to the two first stimulations in the series. Differences between the two first stimulations and the following ones in the series were evaluated using two-way RM ANOVA followed by Sidak’s *post hoc* tests. **p < 0.01 and ***p < 0.001 significantly different.

In the *in vitro* spinal cord preparation of new-born rodents, bouts of fictive locomotion can be induced by 50 Hz stimulation of the VLF and recorded from lumbar ventral roots (Magnuson and Trinder, 1997). We then took advantage of this experimental protocol to decipher the functional impact of mGluR modulation at the motor network level under experimental conditions that should trigger ADSP at VLF-MN synapses. For this purpose, series of 10 stimulations (50 Hz, 2 s) of the VLF were applied every 2 min to induce episodes of fictive locomotion that were compared in the absence and presence of the mGluR agonists. To assess possible differential effects on extensor and flexor MNs, recordings were performed from the L2 and L5 ventral roots (Cazalets et al., 1992; Kiehn and Kjaerulff, 1996). In control aCSF alone, all preparations examined exhibited episodes of evoked fictive locomotion with stable period values (Figures 7A,F), numbers of bursts (6.2 ± 0.6 for the first VLF stimulation and 6 ± 0.5 for the last stimulation, *n* = 14 preparations) and L2 burst amplitudes (Figures 7A,G) across individual series of 10 VLF stimulations, although a small decline in the amplitude of L5 motor bursts occurred (Figures 7A,H). As indicated in the section “MATERIALS AND METHODS,” the three agonists were tested on each spinal cord preparation, with successive applications being separated by a one hour period of aCSF perfusion to allow a complete wash-out of the preceding applied agonist. After each drug trial, effective washout was reached as indicated by the similar mean periods and even larger motor burst amplitudes measured during the two first evoked locomotor episodes in each stimulation series (mean period: aCSF 1.3 ± 0.05 s, DHPG 1.3 ± 0.08 s, LY354740 1.3 ± 0.06 s, L-AP4 1.2 ± 0.05 s, *n* = 14, Kruskal-Wallis test, *p* = 0.63; L2 amplitude relative to that in aCSF: DHPG 1.7 ± 0.3, LY354740 1 ± 0.06, L-AP4 1.3 ± 0.2, *n* = 14, Kruskal-Wallis test, *p* = 0.06; L5 amplitude relative to that in aCSF: DHPG 1 ± 0.1, LY354740 1.5 ± 0.1, L-AP4: 1.5 ± 0.2, *n* = 14, Kruskal-Wallis test, *p* = 0.01, significantly different).

In the presence of DHPG, all preparations tested maintained the ability to express locomotor episodes throughout each stimulation series (Figure 7E). The amplitudes of L2 and L5 bursts remained stable during DHPG application (Figures 7B,G,H and Table 6, L2 and L5 amplitude) but the rhythm cycle period progressively increased until it became significantly different from that in the aCSF condition for the 9th and 10th VLF stimulations (Figures 7B,F and Table 6, period of the VLF-induced activity). In contrast, both LY354740 and L-AP4 led to an eventual suppression of the VLF-induced fictive locomotion before the 10*^th^* stimulation in about 50% of the preparations tested (Figure 7E). In the remaining preparations that still expressed locomotor activity, L-AP4 induced a significant reduction of the rhythm period (Figures 7D,F and Table 6, period of the VLF-induced activity) as well as causing a strong decrease in both L2 and L5 motor burst amplitudes (Figures 7D,G,H and Table 6, L2 and L5 burst amplitude). In contrast, LY354740 only affected locomotor burst amplitudes, but did not significantly alter the cycle period of each evoked locomotor episode (Figures 7C,F,G,H and Table 6).

**TABLE 6:** Results of the Kruskal–Wallis tests performed to analyze the effects of the metabotropic glutamate receptor (mGluR) agonist (drugs) across series of 10 stimulations (stimulation number) applied to the ventrolateral funiculus (VLF) axons on the period of the VLF-induced fictive locomotion as well as lumbar 2 (L2) and L5 motor burst amplitudes.

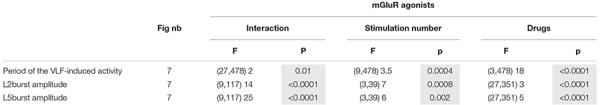

*The numbers into brackets correspond to the degrees of freedom. Fig nb, number of the corresponding Figure. Shaded cells, p < 0.05 significantly different.*

### Modulation of the Swimming Behavior by Metabotropic Glutamate Receptor Agonists

**FIGURE 8:**
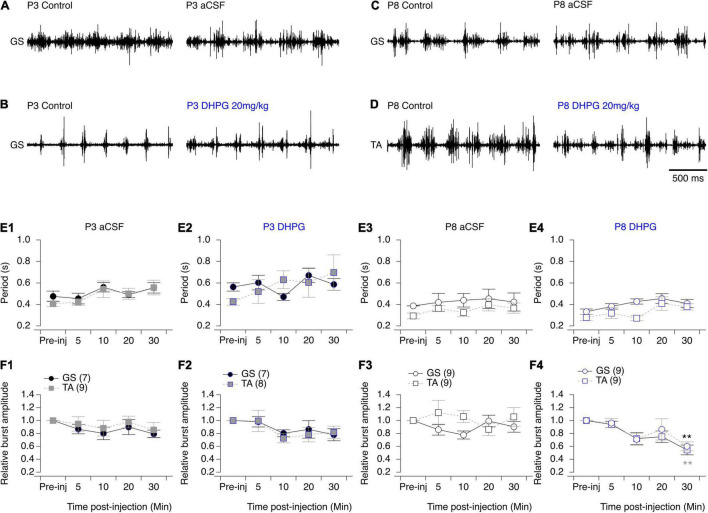
Effects of aCSF and mGluRI agonist on spontaneous swimming activity in mice. **(A–D)** Representative electromyographic recordings from a hindlimb *Gastrocnemius* (GS) or *Tibialis Anterior* (TA) muscle before (control) and 30 min after the injection of aCSF **(A,C)** or DHPG **(B,D)** in P3 **(A,B)** and P8-P10 **(C,D)** mice. **(E)**. Cycle period of the swimming activity recorded from the GS (black circles) or TA (gray squares) before (pre-inj) and at different time points (5, 10, 20, and 30 min) after the injection of aCSF **(E1–E3)** or DHPG **(E2–E4)**. **(F)** Same data representations as in **(E)** for swimming burst amplitudes normalized to mean amplitude values. DHPG induced a significant decrease in the period of the swimming activity in P8 mouse pups. Numbers in brackets correspond to the number of animals tested. Two-way RM ANOVA followed by Sidak’s *post hoc* tests. **p < 0.01 significantly different from the pre-injection burst amplitude.

**FIGURE 9:**
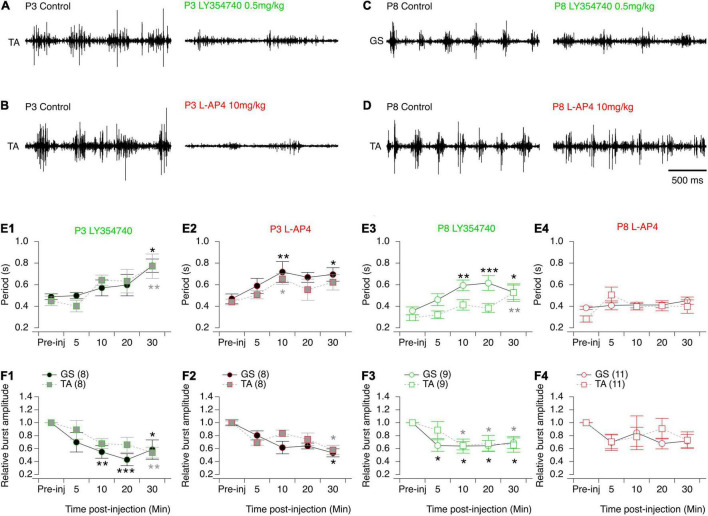
Effects of the mGluRII and III agonists on spontaneous swimming activity in mice. **(A–D)** Representative electromyographic recordings from a hindlimb *Tibialis Anterior* (TA) or *Gastrocnemius* (GS) muscle before (control) and 30 min after the injection of LY354740 **(A–C)** or L-AP4 **(B–D)** in P3 **(A–B)** and P8-P10 **(C–D)** mice. **(E)** Cycle period of the swimming activity recorded from the GS (circles) or TA (squares) before (pre-inj) and at different time points (5, 10, 20, and 30 min) after the injection of LY354740 **(E1–E3)** or L-AP4 **(E2–E4)**. **(F)** Same data representations as in **(E)** for swimming burst amplitudes normalized to the mean amplitude values before mGluR agonist injection. Note that LY354740 and L-AP4 significantly altered both the period and motor burst amplitudes of the swimming activity of P3 mouse pups. In contrast, in P8 animals such effects were only found significant following the injection of LY354740. Numbers into brackets correspond to the number of animals tested. Two-way RM ANOVA followed by Sidak’s *post hoc* tests. *p < 0.05; **p < 0.01 and ***p < 0.001 significantly different from the pre-injection cycle period.

All three mGluR agonists tested have been shown to modulate neuronal processes through systemic routes of administration (Linden et al., 2004; Jin et al., 2012; Zhang et al., 2012). On this basis, together with the relative permeability of the blood brain barrier in newborn rodents (Saunders et al., 2012), we then tested the effects of subcutaneous injections of mGluR agonists on the locomotor behavior of P3 and P8-P10 pups by making EMG recordings from the *Gastrocnemius* and *Tibialis Anterior* muscles during episodes of spontaneous swimming behavior (see Materials and Methods). The subcutaneous injection of aCSF had no effect on the parameters of monitored swimming activity, either of P3 (Figures 8A,E1,F1 and Table 7) or P8-P10 animals (Figures 8C,E3,F3 and Table 7). In P3 mice, DHPG did not modify either swimming cycle periods or the amplitudes of motor bursts recorded from hindlimb muscles (Figures 8B,E2,F2 and Table 7). In P8-P10 animals, however, whereas DPHG was still ineffective in modulating swimming cycle period, a significant decrease in motor burst amplitudes compared to pre-injection values were observed in both recorded muscles (Figures 8D,E4,F4 and Table 7). In contrast, the mGluRII agonist LY354740 exerted similar effects when injected into both P3 or P8-P10 animals, leading to a progressive slowdown of spontaneous swimming activity coupled with a significant decrease in the amplitudes of motor bursts in both muscles recorded (Figures 9A,E1,F1, 9C,E3,F3, respectively and Table 7). Finally, the injection of L-AP4 significantly altered swim cycle period as well as the amplitude of both *Gastrocnemius* and *Tibialis* motor bursts in P3 pups (Figure 9B,E2,F2 and Table 7) but failed to induce significant changes in P8-P10 pups (Figures 9D,E4,F4 and Table 7).

**TABLE 7:** Results of the repeated measure two-way analysis of variances (ANOVAs) performed to test the effects of artificial cerebrospinal fluid (aCSF), 3,5-dihydroxy- phenylglycine (DHPG), LY354740 and L-AP4 at different times after injection (Time after inj) on the period and motor burst amplitudes computed during bouts of swimming activity recorded in the *Gastrocnemius* or *Tibialis* muscles (Muscle) in P3 and P8-P12 mouse pups.

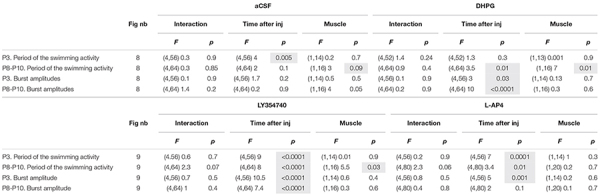

*The numbers into brackets correspond to the degrees of freedom. Fig nb, number of the corresponding Figure. Shaded cells, p < 0.05 significantly different.*

## Discussion

After providing the first evidence that flexor and extensor MNs exhibit different types of ADSP after HFS at VLF-MN synapses (Lenschow et al., 2016), the present study identified a differential modulation of ADSP by glutamate through the activation of mGluRs that is synchronized to a high expression of Grm genes in mouse lumbar MNs during the first postnatal week. Moreover, and to the best of our knowledge, we report for the first time that *Tibialis* and *Gastrocnemius* MNs are characterized by specific molecular profiles with different expression levels of the Grm2 gene in early developmental stages.

The present investigation as well as previous studies, have demonstrated that neither broad spectrum nor specific mGluR antagonists have significant effects *per se* on spinal MNs or VLF-MN synapses (Marchetti et al., 2003; Arvanian et al., 2005; Iwagaki and Miles, 2011) suggesting an absence of endogenous, tonically activated mGluRs. Here, we show that mGluR activation can be induced by increasing synaptic glutamate concentration with the glutamate uptake blocker TBOA or high frequency stimulation of presynaptic axons that revealed the extrasynaptic localization of mGluRs at synapses impinging onto lumbar MNs. This is in agreement with studies elsewhere in the CNS that have described mGluR as being mainly perisynaptic and whose activation requires glutamate spillover from the synaptic cleft (Niswender and Conn, 2010). We observed that the paired pulse ratio was significantly different in the presence of DHPG but unchanged in the presence of LY354741 and L-AP4 at both P1-P3 and P8-P12 VLF-MN synapses. Associated with the results we obtained on MN membrane properties and synaptic transmission, this study further indicates that mGluRII and mGluRIII are located on both the pre- and postsynaptic sides of the VLF-MN synapses and act as inhibitory autoreceptors at the two developmental stages tested. In contrast, mGluRI appear solely present at the postsynaptic level in lumbar MNs. The three mGluR groups thus appear to be colocalized at VLF-MN terminals as previously shown in the lamprey (Krieger et al., 1996; Krieger and Manira, 2002) and exhibit the classical location reported in supraspinal structures (Niswender and Conn, 2010) as well as in the dorsal (Goudet, 2018) and ventral spinal cord (Krieger et al., 1996; Kettunen et al., 2002, 2003; Krieger and Manira, 2002; Iwagaki and Miles, 2011). In the presence of each of the three mGluR agonists, and regardless of age, we observed an increased input resistance and a blockade of the I_*H*_ current. This is in sharp contrast with previous studies that reported a decrease in membrane input resistance coupled with a decrease in Na^2+^ current with DHPG (Iwagaki and Miles, 2011) or an absence of postsynaptic changes in the presence of the mGluRII and GluRIII agonists (Taccola et al., 2004b). The low concentrations of agonists used in our study, which nevertheless appeared very potent in their ability to decrease VLF-MN synaptic transmission, could potentially explain these discrepancies. We observed that the mGluR-mediated modulations of intrinsic membrane properties as well as VLF-EPSC amplitude values and paired pulse ratio were similar whatever the ADSP profile expressed by MNs. These data suggest that all these parameters are similarly modulated in flexor and extensor MNs through mGluR activation (Lenschow et al., 2016).

To assess the neuromodulatory actions of glutamate via mGluRs, we used selective agonists instead of antagonists. This choice was motivated by at least two major factors. First, we could not anticipate the type of plasticity expressed at VLF-MN synapses except when retrogradely labeled MNs from the *Gastrocnemius* or the *Tibialis Anterior* muscles are recorded (Lenschow et al., 2016). And this situation is only valid for P1-P3 mice, since *Gastrocnemius* MNs have been shown to express two different types of responses - LTD or no plasticity - following VLF HFS during the second postnatal week (Lenschow et al., 2016). Recording from identified MNs would have required at least to double the number of experimental animals used, as patch recording from identified neurons is low-yield compared to recording from unidentified MNs. Second, in the presence of the broad spectrum mGluR antagonist MCPG, some P1-P3 VLF-MN synapses devoid of post VLF-HFS modulation were observed, although both LTD and STD were still expressed in these experimental conditions. In contrast, in the presence of an increased mGluR activation with TBOA, we observed a complete disappearance of STD. These findings therefore suggest that mGluRs are involved in ADSP modulation at VLF-MN synapses, but are not mandatory for the expression of LTD and STD. This in turn led us to investigate the impact of mGluR agonists rather than antagonists at the cellular, network and behavioral levels.

In P1-P3 MNs, DHPG did not alter the synaptic transmission at VLF-MN synapses, whereas LY354740 and L-AP4 strongly decreased it, but all three agonists modulated ADSP expression with similar effects occurring under DHPG and L-AP4. Blocking mGluRs or selectively activating the mGluRI or III with DHPG and L-AP4, respectively, both led to the occurrence of LTD, STD and non-plastic VLF-MN synapses after HFS in P1-P3 Mns. These results could not be easily explained. In the present study, we report that the three different subtypes of mGluRs are present at VLF-MN synapses. Depending on their intracellular coupling, mGluRs exert opposite effects in neurons. Moreover, we face a complete lack of insight into the cellular basis of HFS-evoked STD or LTD at these synapses. In P8-P12 MNs, while all three mGluR agonists depressed synaptic transmission, no effect was observed on ADSP. This suggests that changes in ADSP at VLF-MN synapses are not merely the result of a decrease in synaptic release probability. We think that further experiments are required to decipher the precise cellular mechanisms that generate STD and LTD to understand the mGluR-mediated ADSP modulation in MNs.

In P1-P3 MNs, LY354740, as well as TBOA likely through mGluRII activation, selectively abolish the STD that has been shown to be specifically expressed in *Tibialis* MNs (Lenschow et al., 2016). This result is highly consistent with the finding that *Tibialis* MNs appear to express a higher level of the Grm2 gene compared to *Gastrocnemius* MNs. In turn, DHPG and L-AP4 triggered the occurrence of synapses devoid of ADSP at VLF-MN synapses. Consequently, LY354740, and to a lesser extent DHPG and L-AP4, appeared to be promising tools for assessing the functional role of ADSP in spinal motor networks. It is noteworthy, however, that we found similar effects of LY354740 on L2 and L5 motor bursts in the isolated spinal cord preparation, as well as on EMG bursts recorded from *Gastrocnemius* and *Tibialis* muscles during swimming. Moreover, while the actions of LY354740 on ADSP expression are completely different in P1-P3 and P8-P12 MNs, the agonists effects *in vivo* were found to be similar at both developmental stages tested. In a similar manner, DHPG and L-AP4, which have the same effects on ADSP expression, exhibited completely different actions in both the isolated spinal cord preparation and *in vivo*. When tested at the functional level, we observed as others (Taccola et al., 2004a; Chapman and Sillar, 2007; Kyriakatos and Manira, 2007; Iwagaki and Miles, 2011), that mGluR mediated-actions are not restricted to MNs, but are also exerted on interneurons of the CPG circuitry, as indicated by the observed increase in cycle period of VLF-induced activity in the presence of DHPG and L-AP4 and of spontaneous swimming episodes (LY354740 and L-AP4 at P3 and LY354740 at P8-P12). The multiplicity of spinal cellular targets of the metabotropic glutamatergic system thus completely precludes the possibility to selectively decipher the functional impact of ADSP modulation by mGluRs from our current data.

Our RT-qPCR analysis revealed a general decrease in mGluR gene expression during the second postnatal week in the ventral lumbar spinal cord. On the other hand, in P8-P12 mice, a specific upregulation of the Grm3 and Grm7 genes in L5 ventral segments was observed in conjunction with an absence of effects of L-AP4 on swimming cycle period. The locomotor CPG is located in the ventral part of the spinal cord between the lower thoracic and L2 segments (Cazalets et al., 1995). Moreover, it has been shown that premotor interneurons differ between flexor and extensor MNs (Stepien et al., 2010). The difference observed for the Grm3 and Grm7 genes between the L2 and L5 segments could therefore partly rely on a decrease in mGluR expression in CPG neurons and flexor-related premotor interneurons at P8-P12.

In conclusion, our study provides novel insights into the numerous cellular targets of the metabotropic glutamatergic system in the ventral spinal cord, and more especially, on the system’s developmental expression and effects at both the cellular and behavioral levels. The two first postnatal weeks are critical for the maturation of spinal motor networks and behaviors in rodents (Cazalets et al., 1990; Geisler et al., 1993; Vinay et al., 2000, 2002). Our data indicate that during early postnatal life, mGluRs are highly expressed in these circuits and then exhibit changes both in their expression levels and functional impact during the second postnatal week. This in turn suggests that mGluRs might play a key role in the postnatal maturation of motor spinal networks, as previously established for numerous supraspinal structures (Catania et al., 1994).

While the various and complex mGluR-mediated actions prevent us from determining the precise functional role of ADSP expressed at VLF-MN synapses, the fact that these synaptic processes appear to be developmentally modulated by this neuromodulatory system further suggests that synaptic plasticity is likely to make an important contribution to shaping spinal motor networks during postnatal development.

## Data Availability Statement

The original contributions presented in the study are included in the article/supplementary material, further inquiries can be directed to the corresponding author/s.

## Ethics Statement

The animal study was reviewed and approved by University of Bordeaux and the French Agriculture and Forestry Ministry for handling animals (approval number 2016012716035720).

## Author Contributions

SB conceived the study. CQ and SB designed and performed the experiments and analyzed the data. CQ, J-RC, and SB wrote the manuscript. All authors contributed to the article and approved the submitted version.

## Conflict of Interest

The authors declare that the research was conducted in the absence of any commercial or financial relationships that could be construed as a potential conflict of interest.

## Publisher’s Note

All claims expressed in this article are solely those of the authors and do not necessarily represent those of their affiliated organizations, or those of the publisher, the editors and the reviewers. Any product that may be evaluated in this article, or claim that may be made by its manufacturer, is not guaranteed or endorsed by the publisher.

## Abbreviations

aCSF: artificial cerebrospinal fluid
Actß: actineß
ADSP: activity-dependent synaptic plasticity
AHP: after-spike hyperpolarization
AMPA: α -amino-3-hydroxy-5-methyl-4-isoxazolepropionic acid
AP: action potential
cDNA: complementary DNA
CNS: central nervous system
CPG: central pattern generator
CTB: cholera toxin ß-subunit
DHPG: 3,5-dihydroxy- phenylglycine
EMG: electromyography
EPSCs: excitatory postsynaptic currents
Grm: mGluR genes
HFS: high frequency stimulation
L-AP 4: l-2-amino-4-phosphonobutylate
LCM: laser capture microdissection
LTD: long term depression
LY354740: (1S,2S,5R,6S)-2-aminobicyclo[3.1.0]hexane-2,6-dicarboxylic acid
MCPG: methylene cyclopropyl glycine
mGluR: metabotropic glutamate receptor
MNs: motoneurons
mRNA: messenger RNA
NMDA: N-methyl -D-aspartate
PCR: polymerase chain reaction
PPF: paired-pulse facilitation
qPCR: real time PCR
RT-qPCR: real-time reverse transcription polymerase chain reaction
SDHA: succinate dehydrogenase
STD: short-term depression
TBOA: DL-threo-benzyloxyaspartic acid
VLF: ventrolateral funiculus.
